# Supplementary material for: DNA Replication-Transcription Conflicts Do Not Significantly Contribute to Spontaneous Mutations Due to Replication Errors in Escherichia coli
Source: mBio. 2021 Oct 12;12(5):e02503-21. doi: 10.1128/mBio.02503-21 (PMC8510543; doi:10.1128/mBio.02503-21)
Supplement: TABLE S1 [file mbio.02503-21-st001.docx]

**Table S1**. Aggregate gene and mutation data

|  |  | *E. coli* MMR^—^strains | | | |  | *E. coli* MMR^—^ Mfd^—^ strains | | | |
| --- | --- | --- | --- | --- | --- | --- | --- | --- | --- | --- |
| Gene category | No. of CDSs | No. of CDS with BPSs | No. of BPSs in CDSs | No. of CDSs with indels | No. of Indels in CDSs |  | No. of CDS with BPSs | No. of BPSs in CDSs | No. of CDSs with indels | No. of Indels in CDSs |
| All Genes |  |  |  |  |  |  |  |  |  |  |
| All CDSs | 4,511 | 4,133 | 27,164 | 1,497 | 3,841 |  | 2,576 | 4,846 | 530 | 790 |
| CD CDSs | 2,467 | 2,261 | 14,890 | 810 | 2,119 |  | 1,409 | 2,581 | 302 | 426 |
| HO CDSs | 2,044 | 1,872 | 12,274 | 687 | 1,722 |  | 1,167 | 2,265 | 228 | 364 |
| Genes minus tRNA and ribosomal genes | | | | |  |  |  |  |  |  |
| All CDSs | 4,348 | 4,035 | 26,923 | 1,478 | 3,711 |  |  |  |  |  |
| CD CDSs | 2,342 | 2,185 | 14,691 | 803 | 2,083 |  |  |  |  |  |
| HO CDSs | 2,006 | 1,850 | 12,232 | 675 | 1,628 |  |  |  |  |  |
| Highly expressed genes | | |  |  |  |  |  |  |  |  |
| All CDSs | 770 | 674 | 3,688 | 160 | 418 |  | 381 | 665 | 58 | 103 |
| CD CDSs | 483 | 420 | 2,323 | 88 | 195 |  | 241 | 408 | 28 | 45 |
| HO CDSs | 287 | 254 | 1,365 | 72 | 223 |  | 140 | 257 | 30 | 58 |
| Highly expressed genes minus tRNA and ribosomal genes | | | | | |  |  |  |  |  |
| All CDSs | 657 | 594 | 3,486 | 148 | 303 |  |  |  |  |  |
| CD CDSs | 396 | 357 | 2,156 | 86 | 171 |  |  |  |  |  |
| HO CDSs | 261 | 237 | 1,330 | 62 | 132 |  |  |  |  |  |
| Highly expressed genes minus tRNA genes | | | | | |  |  |  |  |  |
| All CDSs | 713 | 643 | 3,633 | 149 | 304 |  | 370 | 651 | 53 | 76 |
| CD CDSs | 447 | 403 | 2,298 | 87 | 172 |  | 236 | 402 | 27 | 40 |
| HO CDSs | 266 | 240 | 1,335 | 62 | 132 |  | 134 | 249 | 26 | 36 |
| Essential genes | | |  |  |  |  |  |  |  |  |
| All CDSs | 358 | 338 | 2,232 | 17 | 31 |  |  |  |  |  |
| CD CDSs | 252 | 242 | 1,605 | 11 | 15 |  |  |  |  |  |
| HO CDSs | 106 | 96 | 627 | 6 | 16 |  |  |  |  |  |
| Ribosomal genes | | |  |  |  |  |  |  |  |  |
| All CDSs | 76 | 53 | 166 | 6 | 13 |  |  |  |  |  |
| CD CDSs | 72 | 51 | 162 | 6 | 13 |  |  |  |  |  |
| HO CDSs | 4 | 2 | 4 | 0 | 0 |  |  |  |  |  |
| *tRNA genes |  |  |  |  |  |  |  |  |  |  |
| All genes | 86 | 44 | 74 | 13 | 117 |  | 14 | 18 | 7 | 29 |
| CD genes | 53 | 25 | 37 | 1 | 23 |  | 6 | 7 | 1 | 5 |
| HO genes | 33 | 19 | 37 | 12 | 94 |  | 8 | 11 | 6 | 24 |
| *tRNA genes not in *rrn* operons | | |  |  |  |  |  |  |  |  |
| All genes | 72 | 38 | 64 | 13 | 117 |  |  |  |  |  |
| CD genes | 39 | 19 | 27 | 1 | 23 |  |  |  |  |  |
| HO genes | 33 | 19 | 37 | 12 | 94 |  |  |  |  |  |
| *tRNA genes minus *leuP,Q,T,V* | | |  |  |  |  |  |  |  |  |
| All genes | 82 | 40 | 56 | 9 | 17 |  | 11 | 14 | 3 | 5 |
| CD genes | 52 | 24 | 36 | 0 | 0 |  | 5 | 6 | 1 | 1 |
| HO genes | 30 | 16 | 20 | 9 | 17 |  | 6 | 8 | 3 | 5 |
| *tRNA genes not in *rrn* operons minus *leuP,Q,T,V* | | | | | | | | |  |  |
| All genes | 68 | 34 | 46 | 9 | 17 |  |  |  |  |  |
| CD genes | 38 | 18 | 26 | 0 | 0 |  |  | | | |
| HO genes | 30 | 16 | 20 | 9 | 17 |  |  |  |  |  |
| All promoters | |  |  |  |  |  |  |  |  |  |
| All genes | 8,568 | 1,050 | 1,235 | 150 | 302 |  |  |  |  |  |
| CD genes | 4,956 | 605 | 709 | 84 | 164 |  |  |  |  |  |
| HO genes | 3,612 | 445 | 526 | 66 | 138 |  |  |  |  |  |
| Promoters of known genes | | |  |  |  |  |  |  |  |  |
| All genes | 3,816 | 477 | 545 | 90 | 167 |  |  |  |  |  |
| CD genes | 2,022 | 251 | 288 | 46 | 80 |  |  |  |  |  |
| HO genes | 1,794 | 226 | 257 | 44 | 87 |  |  |  |  |  |
| Promoters of highly Expressed Genes | | | | |  |  |  |  |  |  |
| All genes | 832 | 101 | 115 | 17 | 38 |  |  |  |  |  |
| CD genes | 469 | 55 | 64 | 8 | 16 |  |  |  |  |  |
| HO genes | 363 | 46 | 51 | 9 | 22 |  |  |  |  |  |
| Promoters of essential genes | | | |  |  |  |  |  |  |  |
| All genes | 289 | 33 | 39 | 6 | 15 |  |  |  |  |  |
| CD genes | 190 | 22 | 24 | 6 | 15 |  |  |  |  |  |
| HO genes | 99 | 11 | 15 | 0 | 0 |  |  |  |  |  |

*Includes *ssrA*, which encodes tmRNA

MMR, mismatch repair; Mfd, mutation frequency decline; CDSs, coding sequences; BPSs, base pair substitutions; Indels, insertions and deletions ≤ 4 bp; CD, codirectional with replication; HO, head-on to replication.
