# Supplementary material for: DNA Replication-Transcription Conflicts Do Not Significantly Contribute to Spontaneous Mutations Due to Replication Errors in Escherichia coli
Source: mBio. 2021 Oct 12;12(5):e02503-21. doi: 10.1128/mBio.02503-21 (PMC8510543; doi:10.1128/mBio.02503-21)
Supplement: TABLE S3 [file mbio.02503-21-st003.docx]

**Table S3**. Comparisons of the slopes of BPSs per CDS vs CDS length in Nts between genes oriented CD versus HO to replication

|  | Slope | SE Slope |  | intercept | SE intercept |  | R^2^ | *P_F_ |
| --- | --- | --- | --- | --- | --- | --- | --- | --- |
| All genes |  |  |  |  |  |  |  |  |
| All genes | 0.0066 | 0.0001 |  | -0.06 | 0.08 |  | 0.65 | <0.0003 |
| CD genes | 0.0066 | 0.0001 |  | -0.07 | 0.11 |  | 0.65 | <0.0003 |
| HO genes | 0.0066 | 0.0001 |  | -0.05 | 0.12 |  | 0.65 | <0.0003 |
| #ΔHO | 0% |  |  |  |  |  |  |  |
| †P | 0.99 |  |  |  |  |  |  |  |
| Genes minus tRNA and ribosomal genes | | | | |  |  |  |  |
| All genes | 0.0068 | 0.0001 |  | -0.20 | 0.08 |  | 0.66 | <0.0003 |
| CD genes | 0.0069 | 0.0001 |  | -0.20 | 0.11 |  | 0.68 | <0.0003 |
| HO genes | 0.0066 | 0.0001 |  | -0.08 | 0.12 |  | 0.65 | <0.0003 |
| #ΔHO | -4% |  |  |  |  |  |  |  |
| †P | 0.10 |  |  |  |  |  |  |  |
| Highly expressed genes | | | |  |  |  |  |  |
| All genes | 0.0073 | 0.0002 |  | -0.17 | 0.14 |  | 0.74 | <0.0003 |
| CD genes | 0.0071 | 0.0002 |  | -0.07 | 0.17 |  | 0.75 | <0.0003 |
| HO genes | 0.0077 | 0.0003 |  | -0.37 | 0.24 |  | 0.72 | <0.0003 |
| #ΔHO | 8% |  |  |  |  |  |  |  |
| †P | 0.15 |  |  |  |  |  |  |  |
| Highly expressed genes minus tRNA and ribosomal genes | | | | | | |  |  |
| All genes | 0.0074 | 0.0002 |  | -0.30 | 0.17 |  | 0.73 | <0.0003 |
| CD genes | 0.0072 | 0.0002 |  | -0.14 | 0.21 |  | 0.74 | <0.0003 |
| HO genes | 0.0079 | 0.0003 |  | -0.59 | 0.27 |  | 0.72 | <0.0003 |
| #ΔHO | 10% |  |  |  |  |  |  |  |
| †P | 0.12 |  |  |  |  |  |  |  |
| Highly expressed genes minus tRNA genes | | | | | | | |  |
| All genes | 0.0074 | 0.0002 |  | -0.27 | 0.16 |  | 0.73 | <0.0003 |
| CD genes | 0.0071 | 0.0002 |  | -0.11 | 0.19 |  | 0.74 | <0.0003 |
| HO genes | 0.0079 | 0.0003 |  | -0.59 | 0.27 |  | 0.72 | <0.0003 |
| #ΔHO | 11% |  |  |  |  |  |  |  |
| †P | 0.08 |  |  |  |  |  |  |  |
| Essential genes | | | | | | | | |
| All genes | 0.0069 | 0.0002 |  | -0.38 | 0.28 |  | 0.70 | <0.0003 |
| CD genes | 0.0067 | 0.0003 |  | -0.20 | 0.33 |  | 0.71 | <0.0003 |
| HO genes | 0.0075 | 0.0005 |  | -0.94 | 0.54 |  | 0.68 | <0.0003 |
| #ΔHO | 12% |  |  |  |  |  |  |  |
| †P | 0.26 |  |  |  |  |  |  |  |
| ‡Ribosomal genes | | | | | | | | |
| All genes | -0.00002 | 0.0003 |  | 2.20 | 0.34 |  | 0.00008 | 0.96 |
| CD genes | -0.00002 | 0.0003 |  | 2.31 | 0.36 |  | 0.0008 | 0.88 |
| HO genes | 0.0073 | 0.015 |  | -0.66 | 3.44 |  | 0.11 | 0.82 |
| #ΔHO | NA |  |  |  |  |  |  |  |
| †P | NA |  |  |  |  |  |  |  |

SE, standard error of the estimate of the parameter.

R^2^, the coefficient of determination, which is the fraction of the variation of the variable, in this case BPSs per CDS, that is explained by the linear model.

*P_F_, the probability that the regression occurred by chance, calculated from the F distribution (22) and adjusted for multiple comparisons by the Benjamini–Hochberg procedure (23).

#ΔHO, the % increase of the slope of the HO genes over the slope of the CD genes.

†P, the probability that the slopes for CD and HO oriented genes are equal calculated from the two-tailed Student's t distribution (22) and adjusted for multiple comparisons by the Benjamini–Hochberg procedure (23).

‡Included are all genes for ribosomal RNAs and proteins; excluded are all tRNA genes whether or not they are in *rrn* operons. None of the three slopes is different than zero, plus there are only four ribosomal genes in the HO orientation and their CDSs accumulated only 4 BPSs.

CDSs, coding sequences; BPSs, base pair substitutions; indels, insertions and deletions ≤ 4 bp; CD, codirectional with replication; HO, head-on to replication; NA, not applicable (too few mutations).
