# Supplementary material for: DNA Replication-Transcription Conflicts Do Not Significantly Contribute to Spontaneous Mutations Due to Replication Errors in Escherichia coli
Source: mBio. 2021 Oct 12;12(5):e02503-21. doi: 10.1128/mBio.02503-21 (PMC8510543; doi:10.1128/mBio.02503-21)
Supplement: TABLE S4 [file mbio.02503-21-st004.docx]

**Table S4.** Comparisons of the frequencies of mutations in the promoters of genes oriented CD versus HO to replication

|  |  |  | BPSs/promoter | |  | Indels/promoter | |
| --- | --- | --- | --- | --- | --- | --- | --- |
|  |  |  | Mean | SD |  | Mean | SD |
| All promoters | | | | | | | |
| All genes | |  | 0.14 | 0.42 |  | 0.04 | 0.41 |
| CD genes | |  | 0.14 | 0.41 |  | 0.03 | 0.36 |
| HO genes | |  | 0.15 | 0.42 |  | 0.04 | 0.47 |
| *P | |  | 0.86 |  |  | 0.71 |  |
| Promoters of known genes | | | | | | | |
| All genes | |  | 0.14 | 0.40 |  | 0.04 | 0.39 |
| CD genes | |  | 0.14 | 0.41 |  | 0.04 | 0.33 |
| HO genes | |  | 0.14 | 0.40 |  | 0.05 | 0.45 |
| *P | |  | 0.96 |  |  | 0.62 |  |
| Promoters of highly expressed Genes | | | | | | | |
| All genes | |  | 0.14 | 0.14 |  | 0.05 | 0.48 |
| CD genes | |  | 0.14 | 0.14 |  | 0.03 | 0.31 |
| HO genes | |  | 0.14 | 0.14 |  | 0.06 | 0.63 |
| *P | |  | 0.92 |  |  | 0.59 |  |
| Promoters of essential genes | | | | | | | |
| All genes | |  | 0.13 | 0.40 |  | 0.05 | 0.47 |
| CD genes | |  | 0.13 | 0.36 |  | 0.08 | 0.58 |
| HO genes | |  | 0.15 | 0.46 |  | < 0.01 | NA |
| *P | |  | 0.76 |  |  | NA |  |

*P, the probability that the indicated values for CD and HO oriented genes are equal calculated from the Student's two tailed t distribution (22) and adjusted for multiple comparisons by the Benjamini–Hochberg procedure (23). None of the comparisons are statistically significant with or without the adjustment.

BPSs, base pair substitutions; indels, insertions and deletions ≤ 4 bp; CD, codirectional with replication; HO, head-on to replication; SD, Standard deviation; NA, not applicable (no mutations).
