# Supplementary material for: DNA Replication-Transcription Conflicts Do Not Significantly Contribute to Spontaneous Mutations Due to Replication Errors in Escherichia coli
Source: mBio. 2021 Oct 12;12(5):e02503-21. doi: 10.1128/mBio.02503-21 (PMC8510543; doi:10.1128/mBio.02503-21)
Supplement: TEXT S1 [file mbio.02503-21-s0001.docx]

**Text S1**: Base-pair substitution biases in tRNA genes could be due to biased target positions

The four tRNA *leu* homologues accounted for 18 of the 74 BPSs that accumulated in tRNA genes, which was 6-fold greater than the average frequency in the other 82 tRNA genes (Table S1). In contrast to the indels, the frequencies of these 18 BPSs were biased: *leuP*, *leuQ*, and *leuV*, the three genes-oriented HO to replication, accumulated 6, 4, and 7 BPSs, but *leuT*, the gene oriented CD to replication, accumulated only 1 BPS. This result suggests that there is an influence of transcription direction on the production of BPSs. However, consideration of the types of BPSs suggests an alternative explanation. Twelve of the BPSs, 4 in *leuP*, 3 in *leuQ,* and 5 in *leuV*, were transitions at the A:T bp adjacent to the run of 8 G:C bps (Fig. S3A ). BPSs adjacent to runs can be ascribed to loop-out of the primer during DNA synthesis, synthesis of an extra base, then realignment to the template, creating a mispair with the base 3′ to the run (19,60). This could occur during leading strand replication in *leuP*, *Q*, and *V*, but only during lagging strand replication in *leuT*. If we assume that primer loop-out is much more likely during leading strand replication than during lagging strand replication, the mutational bias is explained. Supporting this hypothesis, the one BPS in *leuT*, an A:T to C:G transversion that occurred at the A:T bp adjacent to the 5 G:C bp run could be made during leading strand replication in *leuT*, but did not occur in *leuP*, *Q*, or *V*, where it could only be made during lagging strand replication. (Fig. S3B)

One addition note about these special mutations. One transition of the terminal G:C of the 8 G:C bp run occurred in *leuP*. This mutation could be made by loop-out of the template during lagging strand replication in *leuP*, *Q*, and *V*, but only during leading strand replication in *leuT* (Fig. S3C). So, it is possible that primer loop-out is more frequent during leading strand replication and template loop-out is more frequent during lagging strand replication.
