# Supplementary material for: DNA Replication-Transcription Conflicts Do Not Significantly Contribute to Spontaneous Mutations Due to Replication Errors in Escherichia coli
Source: mBio. 2021 Oct 12;12(5):e02503-21. doi: 10.1128/mBio.02503-21 (PMC8510543; doi:10.1128/mBio.02503-21)
Supplement: TABLE S5 [file mbio.02503-21-st005.docx]

**Table S5**. *Bacillus subtilis* mutational data and results

A. Comparison of the mutation results from genes oriented HO versus CD to replication

|  | No. CDSs | No. CDSs with BPSs | No. BPSs in CDSs | No. CDSs with indels | No. Indels in CDS |  | BPSs per CDS | |  | BPSs/CDS/Nt x 10^3^ | |  | Indels per CDS | |  | Indels/CDS/Nt x 10^3^ | |
| --- | --- | --- | --- | --- | --- | --- | --- | --- | --- | --- | --- | --- | --- | --- | --- | --- | --- |
|  |  |  |  |  |  |  | Mean | SD |  | Mean | SD |  | Mean | SD |  | Mean | SD |
| All genes | 4,418 | 3,281 | 9,587 | 1,118 | 2,100 |  | 2.23 | 2.60 |  | 2.56 | 2.42 |  | 0.48 | 1.09 |  | 0.67 | 1.97 |
| CD genes | 3,254 | 2,427 | 7,049 | 786 | 1,476 |  | 2.17 | 2.61 |  | 2.45 | 2.42 |  | 0.45 | 1.08 |  | 0.57 | 1.65 |
| HO genes | 1,164 | 854 | 2,538 | 332 | 624 |  | 2.18 | 2.52 |  | 2.60 | 2.43 |  | 0.54 | 1.12 |  | 0.92 | 2.65 |
| *ΔHO |  |  |  |  |  |  | 1% |  |  | 6% |  |  | 18% |  |  | 61% |  |
| ^#^P |  |  |  |  |  |  | 0.92 |  |  | 0.15 |  |  | 0.07 |  |  | <0.0003 |  |

B. Correlation of the numbers of BPSs per CDS with the length of the CDS in Nt.

|  | Slope | SE | Intercept | SE | R^2^ | ^†^P_F_ |
| --- | --- | --- | --- | --- | --- | --- |
| All genes | 0.0025 | 3.1 x 10^-5^ | 0.03 | 0.04 | 0.59 | <0.0003 |
| CD genes | 0.0024 | 3.5 x 10^-5^ | 0.03 | 0.04 | 0.59 | <0.0003 |
| HO genes | 0.0027 | 6.7 x 10^-5^ | 0.01 | 0.07 | 0.58 | <0.0003 |
| *ΔHO | 10% |  |  |  |  |  |
| ^#^P | 0.003 |  |  |  |  |  |

*ΔHO, the % increase of the values for the HO genes over those of the CD genes.

^#^P, the probability that the indicated values for CD and HO oriented genes are equal from the two-tailed Student's t distribution (22) and adjusted for multiple comparisons by the Benjamini–Hochberg procedure (23). By the nonparametric Mann-Whitney test, the difference between indels in CDSs oriented HO versus CD is statistically significant, P= 0.01 (adjusted).

R^2^, the coefficient of determination, which is the fraction of the variation of the variable, in this case BPSs per CDS, that is explained by the linear model.

^†^P_F,_ the probability that the regression occurred by chance, calculated from the F distribution and adjusted for multiple comparisons by the Benjamini–Hochberg procedure (23).

CDSs, coding sequences, BPSs, base pair substitutions; indels, insertions and deletions ≤ 4 bp; CD, codirectional with replication; HO, head-on to replication; SD, standard deviation; SE, standard error.
